# Biogenesis of *Candida glabrata*-Mediated Silver Nanoparticles: Characterization and Antibacterial Effectiveness Against Human Pathogenic Bacteria

**DOI:** 10.3390/ijms27031263

**Published:** 2026-01-27

**Authors:** Syed Fahad Akbar Ali, Suhaib Masroor, Muhammad Kashif Shaikh, Gul Jabeen, Sehar Afshan Naz, Afsheen Aqeel, Komal Anjum

**Affiliations:** 1 Department of Biomedical Engineering, Sir Syed University of Engineering and Technology, Gulshan-e-Iqbal, Karachi 75300, Pakistan; sfaali@ssuet.edu.pk; 2 Department of Computer Science and Information Technology, Sir Syed University of Engineering and Technology, Gulshan-e-Iqbal, Karachi 75300, Pakistan; mkshaikh@ssuet.edu.pk; 3 Department of Microbiology, University of Karachi, University Road, Karachi 75270, Pakistan; guljabeen010@gmail.com (G.J.); sehar.afshan@uok.edu.pk (S.A.N.); afsheenaqeel@gmail.com (A.A.); 4 Department of Medicine and Pharmacy, Ocean University of China, Qingdao 266000, China

**Keywords:** antibacterial, biogenesis, *Candida glabrata*, characterization, nanotechnology, silver nanoparticles

## Abstract

In recent years, silver nanoparticles have emerged as potent antimicrobial agents capable of combating extensively drug-resistant pathogenic bacteria that pose serious health risks. The primary aim of our research was to explore the green synthesis of AgNPs using *Candida glabrata* as an antibacterial agent. A single clinical isolate of *Candida glabrata* was re-examined via traditional yeast identification methods. Biosynthesis of AgNPs was accomplished by incubating *Candida glabrata* cell-free supernatant with silver nitrate. AgNP formation was verified by UV-Vis spectroscopy, and the XRD technique assessed the physical properties of the lyophilized AgNPs. EDX and SEM provided insights into the AgNPs’ composition, shape, and size. The antibacterial efficacy was evaluated against pathogenic bacteria through the Agar Well Diffusion method. The formation of AgNPs was evidenced by a shift in color to dark brown. The formation of AgNPs at an absorbance wavelength of 430 nm revealed a polycrystalline structure with an average crystal size of 21.91 nm. The silver constituted 29.50% of the composition and indicated a spherical shape with sizes ranging from 74.96 to 100.40 nm. Significant antimicrobial activity was obtained against pathogenic bacteria. Hence, the proposed research highlights a single-step, cost-effective, and environmentally friendly AgNP synthesis approach that exhibits considerable antibacterial properties.

## 1. Introduction

Nanotechnology has emerged as one of the most researched and successful areas due to its benefits and uses in various aspects of human well-being [1]. It is the study deals with nanoparticles (NPs) at the atomic and molecular scales, with sizes ranging from 1 to 100 nm [2]. Through the construction, synthesis, and manipulation of large molecules and particles at the nanoscale, material science has adopted nanotechnology as an essential means to address various limitations across multiple disciplines [3]. In recent years, nanotechnology has developed as a research area that has helped in creating innovative agents with distinct and favorable traits to combat a wide range of pathogens [4]. In general, the fabrication of NPs involves three primary techniques—physical, chemical, and biological methods—which are employed to create NPs with diverse shapes, sizes, forms, and characteristics [5,6].

The synthesis of NPs through physical and chemical processes comes with multiple drawbacks, including the high costs associated with physical methods and the reliance on hazardous chemicals and reducing agents in chemical methods, which result in toxic by-products that can harm the environment [7]. This highlights the urgent need for a safe, eco-friendly, and economically viable approach to the large-scale biosynthesis of NPs. As a result, research has shifted towards a novel, convenient, environmentally friendly, high-yield, and affordable technique known as green or biological synthesis [8,9]. Consequently, green production provides a practical substitute for traditional chemical and physical methods because it utilizes nontoxic solvents and sustainable materials, which are important in this environmentally friendly approach. The concept of green synthesis involves the creation of NPs from different metals by utilizing the reducing properties of biologically active substances. These biologically active substances can be obtained from microorganisms, such as bacteria, algae, and fungi, as well as from herbal extracts (including leaves, roots, flowers, fruits, bark, and latex), and animal extracts [10].

The production of NPs through fungal biosynthesis exhibits good monodispersity and stability compared to other microorganisms [11]. As fungi can produce larger quantities of NPs than plants and bacteria, they are considered more suitable for NP production. Numerous studies have reported the ability of fungi to synthesize NPs, with the earliest instance being the synthesis of Cadmium selenide (CdSe) NPs by *Candida albicans* in 1989. Since that time, various other filamentous fungi such as *Trichoderma asperellum*, *Cladosporium cladosporioides*, *Fusarium* spp., and *Aspergillus* spp. have been identified as capable of NP synthesis [12].

Generally, different metals, including Gold (Au), Iron (Fe), Cadmium (Cd), Copper (Cu), Nickel (Ni), Selenium (Se), and Platinum (Pt), have been utilized in the biosynthesis of NPs [13]. Among the different types of metals, silver nanoparticles (AgNPs) have recently been the focus of extensive research due to their various physical, chemical, and biological properties that vary in size, shape, function, crystallinity, and structure. Numerous studies are underway to incorporate AgNPs into clinical and industrial applications, along with other technological uses in healthcare [14].

In recent times, AgNPs have gained recognition not only for their antibacterial properties but also as promising options for drug delivery systems, firmly establishing their role in the healthcare sector, including areas such as medicine, catheter modification, dental implants, wound dressings, and bone repair, while also being utilized to enhance the textile and food industries [15,16]. AgNPs can be a potential substitution for conventional antibiotics, particularly with consideration of increasing antimicrobial resistance, since they have more than one simultaneous mechanism of antimicrobial action, so bacteria are less likely to survive only through simple single-genome mutations [17]. They possess a wide spectrum of action on both Gram-negative and Gram-positive bacteria, such as multidrug-resistant (MDR) strains, being no more sensitive to traditional antibiotics [15].

Biosynthesis of AgNPs through the yeast (unicellular fungi)-assisted route is considered to be an attractive green alternative for this purpose as it manifests rapid growth in low nutritional conditions, uniform biomass, and secretion of extracellular enzymes and reductive proteins able to reduce positively charged silver (Ag^+^) ions into relatively homogeneous, colloidally stable monodisperse AgNPs, which are recoverable from the supernatant without having to carry out laborious harvesting steps involving complex disruption of the cellular biomass structure [18]. However, filamentous fungi frequently form mycelial mats or dense hyphal masses, which may entrap the AgNPs and complicate the separation and purification of particles. The heterogeneity of hyphal structures and their physiological conditions may result in discrepancies in the size distribution and batch-to-batch variability of AgNP properties. Filamentous development may also demand more elaborate cultivation conditions (stirring, oxygenation) and longer incubation times, making scaling-up problematic. These observations have been described as major problems in some fungal biosynthesis studies [19].

The capacity for biosynthesizing AgNPs from various fungal isolates differs, as not all fungi facilitate the reduction of silver (Ag) ions [20]. Nonetheless, various studies have documented the biosynthesis of AgNPs using unicellular yeast like *Candida albicans*, *Saccharomyces boulardii*, *Saccharomyces cerevisiae*, *Candida utilis*, and *Candida lusitaniae*; however, many are still rarely investigated. Moreover, *Candida glabrata* (*C. glabrata*) stands out as a particularly promising single-celled yeast for the production of AgNPs through the interaction between Ag ions and secondary metabolites [7]. Use of cell-free supernatants (CFS) of *C. glabrata* strains or their enzymatic extract for the synthesis of AgNPs has a double justification: (1) the secretome of yeast (a set of extracellular enzymes, proteins, and metabolites) can be involved in efficient extracellular reduction and stabilization of Ag ions, leading to AgNPs displaying unique surface coronas and thus biological properties; and (2) NPs synthesized by yeast having relevance in clinic can be easily tested directly against *Candida* spp. and other human pathogens to determine whether the biosynthesis pathway results in particles having improved anti-biofilm or anti-resistance properties. There have been few studies demonstrating one-step extracellular AgNP synthesis using *C. glabrata* isolates that have reported antimicrobial activity against clinical strains, proving feasibility but also bringing into view the need for more rigorous characterization, reproducibility testing, and comparison to other fungal synthesis paths [7,21].

In the current landscape, the emergence of new and resistant pathogenic bacteria presents a significant danger to public health worldwide. As a result, our study concentrated on biologically produced AgNPs using the CFS of a single *C. glabrata* strain, providing a comprehensive microscopic and spectroscopic evaluation, and investigating their potential uses against different pathogenic bacteria to study their antibacterial efficiency. Further, by using the yeast strain under stable experimental conditions, this study identifies the biochemical parameters that are associated with successful AgNP formation and discusses key knowledge gaps outlined in earlier studies regarding the assessment of the structure, morphology, composition, and surface chemistry of AgNPs. Therefore, the present study contributes not only to yeast-mediated AgNP biosynthesis but also to the identification of AgNP-related strain specificity for opportunistic pathogens.

## 2. Results

### 2.1. Validation of C. glabrata Strain

**Figure 1:**
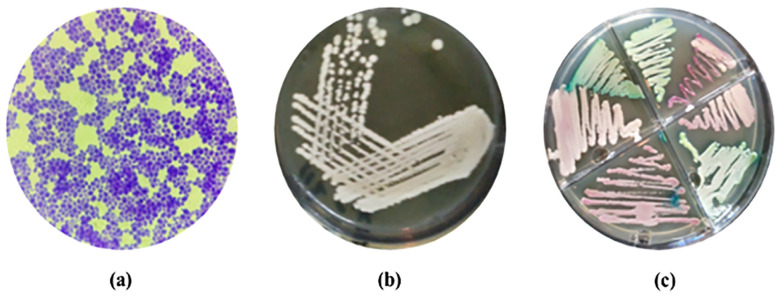
Morphological features of *C. glabrata* isolate: (**a**) microscopic characteristics; (**b**) growth characteristics in SDA; (**c**) growth characteristics in CHROMagar.

The *C. glabrata* strain (FCG-8) was reidentified by conventional diagnostic protocols, as shown in Table 1. The collected strain after Gram staining appeared to have large oval to spherical budding yeast cells due to the retention of crystal violet and produced large, round, smooth, glabrous, and white to cream-colored colonies when viewed macroscopically on Sabouraud Dextrose agar (SDA). It formed white-colored colonies on Chromogenic agar (CHROMagar) (Figure 1). The strain produced a germ tube when inoculated in serum and incubated at 37 °C [22]. 

### 2.2. Confirmation of AgNP Biosynthesis

**Figure 2:**
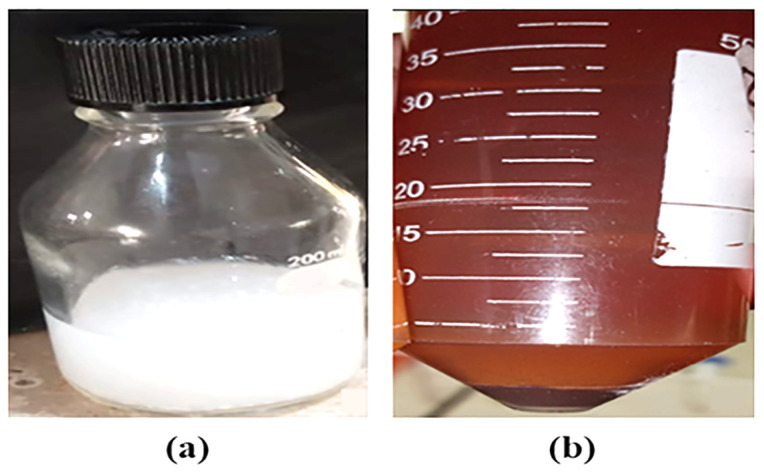
Biosynthesis of AgNPs through CFS of *C. glabrata* isolate: (**a**) before incubation; (**b**) after 48 h at 37 °C in a dark environment.

The formation of AgNPs through biosynthesis was achieved using the CFS solution of a *C. glabrata* strain, where a color transformation from transparent to dark brown was monitored (Figure 2).

### 2.3. UV-Vis Spectroscopy Analysis

**Figure 3:**
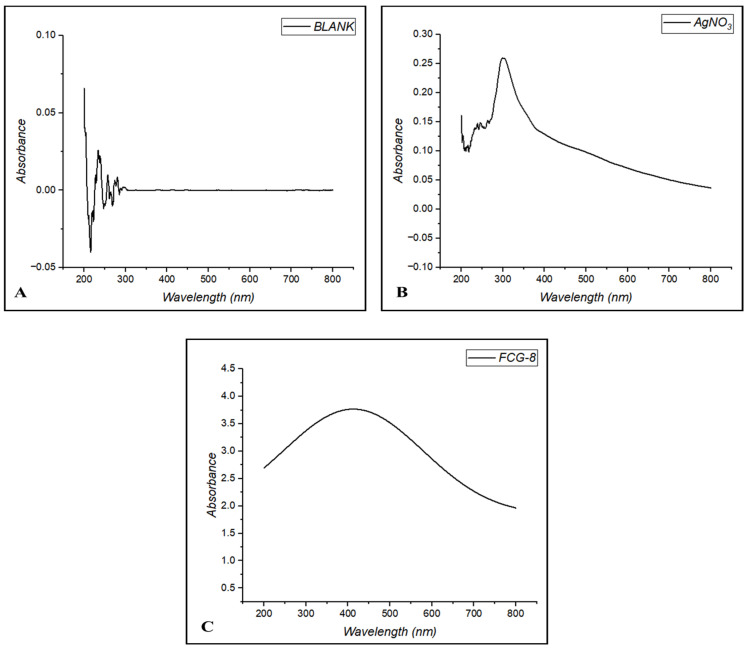
A graphical illustration of ultraviolet–visible spectroscopic analysis: (**A**) blank solution; (**B**) AgNO_3_ solution; (**C**) biosynthesized AgNPs obtained after incubation of the FCG-8 culture filtrate with AgNO_3_.

The UV-Vis analysis for the blank solution, silver nitrate (AgNO_3_) solution, and *C. glabrata* strain (FCG-8) was observed for AgNP characterization (Figure 3). According to the analysis, FCG-8 confirmed the production of AgNPs, displaying an absorbance reading of 3.80 at a wavelength of 430 nm. Conversely, this distinct peak was absent in any of the other samples. The absorbance measured at the maximum wavelength (λmax), ranging from 400 nm to 500 nm, corresponds to the ultraviolet absorption spectrum typical of AgNPs. 

### 2.4. The XRD Analysis

**Figure 4:**
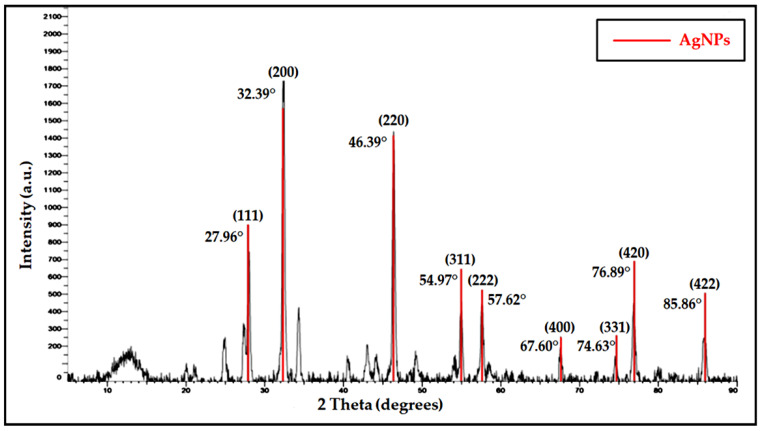
Characterization of AgNPs from FCG-8, using XRD.

After the confirmation of AgNP biosynthesis from FCG-8, the sample was utilized for further characterization using XRD techniques. The complete peak details for FCG-8 were acquired through the XRD analysis, shown in Figure 4. These peaks, (111), (200), (220), (311), (222), (400), (331), (420), and (422), were observed at corresponding angles of 27.96°, 32.39°, 46.39°, 54.97°, 57.62°, 67.60°, 74.63°, 76.89°, and 85.86°. 

### 2.5. The EDX Analysis

**Figure 5:**
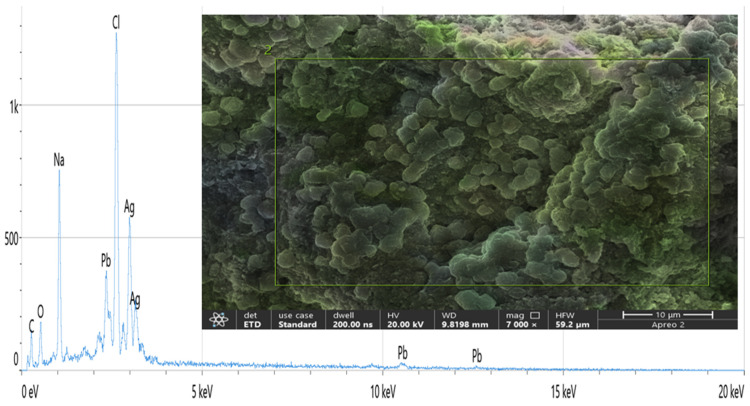
Characterization of the biosynthesized AgNPs using EDX for FCG-8. A graphical representation of the Ag alongside other metals and a micrograph that illustrates the presence of Ag within the overall sample of FCG-8 are shown.

The EDX indicated compositional analysis and verification of the Ag from FCG-8 (Figure 5). The EDX analysis was performed at a 20 kiloelectron volt (keV) accelerating voltage and showed the weight percentage of Ag in the sample to be 29.5 percent (%). Simultaneously, some other elements also appeared through EDX analysis, and their weight fractions are shown in Table 2.

### 2.6. The SEM Analysis

**Figure 6:**
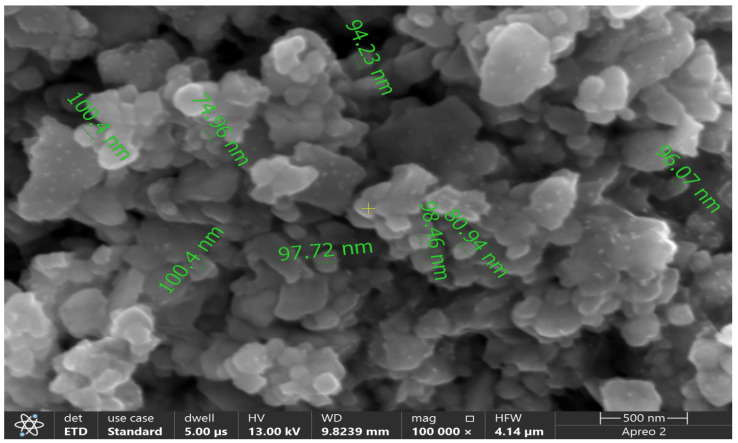
The examination of biosynthesized AgNPs, assessed via SEM scan at 500 nm, showing AgNP sizes from 74.96 to 100.40 nm.

**Figure 7:**
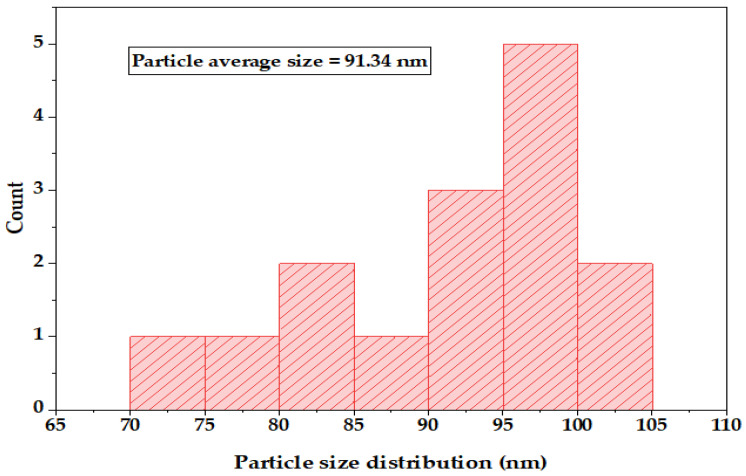
Particle size distribution histogram of AgNPs synthesized by the FCG-8 strain, calculated from SEM micrographs shown in Figure 6, displaying an average particle size of 91.34 ± 2.26 nm.

The spherical shape and size of AgNPs (ranging from 74.96 to 100.40 nm), synthesized using the strain of *C. glabrata* (FCG-8), were analyzed by SEM. The representative SEM micrograph of the sample (FCG-8) is presented in Figure 6 and was used for particle size analysis to enhance the accuracy and reproducibility of the measurements. The individual AgNP diameter was measured by ImageJ software (version 1.53a), and the obtained data were compiled to obtain a histogram of particle size distribution (Figure 7). The SEM analysis was performed by measuring the size of 15 individual NPs, and the histogram shows that most of the synthesized AgNPs lie within the nanoscale range, with an average particle size of 91.34 ± 2.26 nm (mean ± standard error of the mean). The fairly narrow size distribution is indicative of reasonably homogeneous AgNP formation, while the minor size inhomogeneities may be related to particle aggregation and differences in the growth kinetics of AgNPs during the biosynthesis process.

### 2.7. Antibacterial Potentials of Biosynthesized AgNPs

The antibacterial efficacy of AgNPs biosynthesized from the *C. glabrata* strain (FCG-8) was evaluated based on their antibacterial potential compared to the CFS of the respective strain on clinical pathogens, as performed using the Agar Well Diffusion method (normal saline and Cefadroxil as negative and positive controls, respectively), as represented in Table 3. All of the antibacterial experiments were performed in triplicate (n = 3), and results are expressed as mean ± standard error of the mean. The statistical analysis was carried out using an independent two-tailed Student’s *t*-test to compare the antibacterial activity of AgNPs with CFS, where *p*-values < 0.05 were regarded as being statistically significant with respect to all tested pathogens.

**Figure 8:**
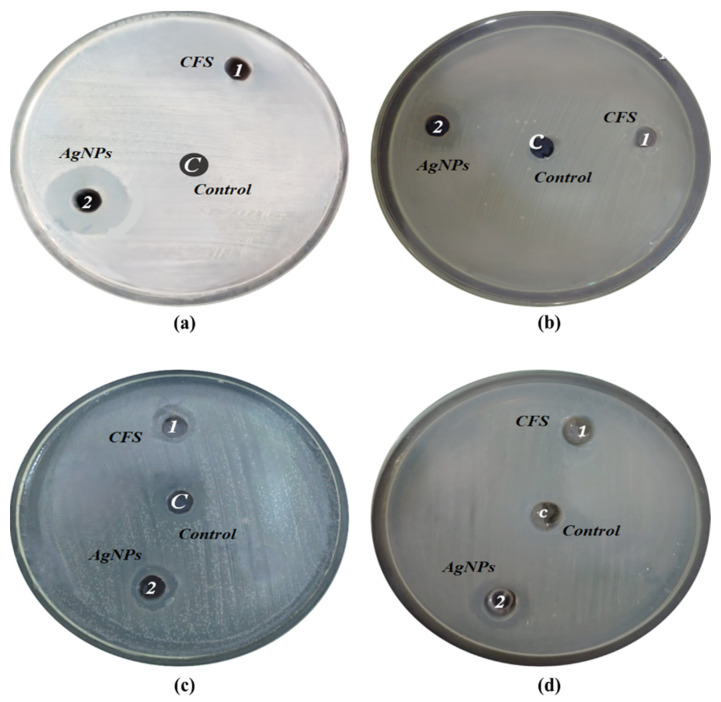
Antibacterial properties of biosynthesized AgNPs and only the CFS solution of FCG-8 against (**a**) *E. coli ATCC^®^ 25922*; (**b**) *S. dysenteriae*; (**c**) *A. hydrophila*; (**d**) *E. fecalis*. Here, well 1 was designated as the zone of inhibition by CFS of FCG-8, well 2 represented the zone of inhibition by biosynthesized AgNPs of FCG-8, and well C showed the negative control (0.9% saline).

The antimicrobial activity data revealed clear differences between the CFS, biosynthesized AgNPs (Figure 8), and the Cefadroxil. The CFS did not show any trace of inhibitive action against *E. coli ATCC^®^ 25922* and *S. dysenteriae*, while for *A. hydrophila*, it caused 7.7 ± 0.33 mm, and for *E. faecalis*, it produced 5.7 ± 0.66 mm of moderate inhibition. Significantly, AgNPs synthesized by FCG-8 exhibited enhanced and broader-spectrum antibacterial activity compared to the use of CFS alone. AgNPs were able to generate inhibition zones against all tested pathogens, which include *E. coli ATCC^®^ 25922* at 18.7 ± 0.66 mm, *S. dysenteriae* at 13.0 ± 1.00 mm, *A. hydrophila* at 10.7 ± 0.66 mm, and *E. fecalis* at 13.3 ± 0.33. Hence, the AgNPs enhanced antibacterial activity by 39% against *A. hydrophila* and 133% against *E. faecalis*, while inducing inhibition in strains where CFS showed no activity. However, Cefadroxil consistently produced larger inhibition zones across all pathogens, suggesting that even though the biosynthesized AgNPs are effective, their antibacterial activity remains inferior compared to a conventional antibiotic under the tested conditions.

## 3. Discussion

As a novel approach, nanotechnology has found immense potential for the development of next-generation antibacterial material, especially to combat the rising problem of MDR pathogens. Metal and metal-based NPs have shown remarkable benefits over traditional antibiotics due to their high surface area, ability to manipulate their physicochemical characteristics, and multiple modes of action that include cell membrane disruption, reactive oxygen species (ROS) formation, and interference with intracellular processes. There has been immense focus on overcoming the problem of biofilms caused by MDR microbes using NPs, emphasizing their potential to overcome diffusion limitations for improving localized antibacterial action [23]. In this scenario, the AgNPs designed for the presented research have shown their relevance to the aforementioned nanotechnology-based approaches meant to enhance antibacterial activity to a greater extent than using biologically designed NPs.

Because of the reduction of Ag^+^ ions in Ag^0^, a color change from transparent to darkish brown was observed, which was attributed to the formation of AgNPs. The plasmonic surface resonance found in AgNPs encourages color variation during the synthesis reaction (reduction of Ag^+^) in the presence of AgNO_3_. The AgNPs within the suspension also had an impact, as the larger or aggregated AgNPs typically have peaks at a longer wavelength than the smaller AgNPs present with the darker coloration [20].

The biosynthesis of AgNPs using *C. glabrata* isolate (FCG-8) was indicated by a change in color, and UV-Vis spectroscopy confirmed the peak absorption wavelength of FCG-8 at 430 nm, which effectively verifies the formation of AgNPs. This phenomenon is attributed to the oscillation of electrons at the boundary between metals and their dielectric medium within the ultraviolet range [24].

The physicochemical properties of biosynthesized AgNPs were detected in FCG-8 at multiple peaks, as determined through XRD investigations. Matched with the ICDD-JCPDS standard Card No. 00-04-0783, the XRD analysis showed peaks at 27.96°, 32.39°, 46.39°, 54.97°,57.62°, 67.60°, 74.63°, 76.89°, and 85.86° at 2θ (degree), associated with AgNP presence and corresponding to the (111), (200), (220), (311), (222), (400), (331), (420), and (422) planes [25,26].

Furthermore, the XRD analysis in our study also revealed the nature of the biosynthesized AgNPs as crystals, with a size of 21.91 nm, as determined by employing the Debye–Scherrer equation. This size falls squarely within the range commonly reported by Velmurugan et al., Sherpa et al., and Thiruvengadam & Bansod in AgNPs synthesized through biogenic routes, which often fall within the ~12–24 nm range of crystallite sizes [25,27,28].

Moreover, Hikmet and Hossein revealed a crystal size of 58 nm in their XRD analysis, which was approximately more than double the size of the crystals obtained in the proposed study [29], which may be because of the variation in temperature, nature of the strain, and biomolecular residues in the filtration process. However, the XRD pattern in the present study revealed that the indexed reflections belong to the powder diffraction pattern of a face-centered cubic (FCC) Ag, thereby confirming crystalline Ag. Although XRD itself does not represent the external shape of particles directly, the relative enhancement in the intensity of reflection (200) compared to standard powder values shows the preferential exposure of facets also as an indication of cubic structure, as Ahmed et al., Ali et al., and Majeed Khan et al. mentioned in their studies [30,31,32].

The EDX assessment involved recognizing the qualitative and quantitative growth of elements in the formation of NPs. The EDX analyses of the FCG-8 sample showed that the highest counts of Ag emerged at the 3 keV optical absorbance band [33], and the weight fraction of Ag was identified as 29.5%. This result is more elevated compared to the Ag weight measured by Hashmi et al., who determined an Ag content of 15.25% in their research [34].

In contrast, our study indicated a much lower weight fraction of Ag when compared to the results obtained by Hikmet & Hossein and Dhabalia et al., who measured the Ag composition at around 82–86% in their EDX analysis by using *Candida albicans* for AgNP creation [29,35]. We attribute the lower Ag content in our sample to residual organic matter from the yeast supernatant, consisting of proteins, polysaccharides, and other biomolecules that act as reducing and capping agents, and to the minimal purification steps [21].

Further, El-Naggar et al. obtained a 34.35% weight fraction for the Ag sample [36], which is quite close to the Ag weight achieved in our study. This observation underlines the fact that in yeast-mediated AgNP synthesis, a lower apparent Ag content is not an exception, and overall, AgNP characteristics depend not only on the Ag mass fraction but also on associated capping biomolecules conferring stability and biological activity. In addition to that, the EDX analysis in our study also indicated the existence of some other elements such as C, O, Na, Cl, and Pb, which were possibly evolved through biological materials originating from the surface of the tested sample [37].

The SEM examination revealed the spherical shape of the biosynthesized AgNPs [38] and size in the 75 to 100 nm range. The average size of the AgNPs determined in our study was significantly larger than the range reported by Mohanta et al., who found AgNPs measuring between 20 and 30 nm [39]. Additionally, the AgNPs in our research exhibit characteristics closer to those described by Ali et al., where the sizes were reported to be between 50 and 70 nm, displaying both spherical and varied shapes [40].

The results of the independent two-tailed Student’s *t*-test in the present study demonstrate that biosynthesized AgNPs significantly outperform crude fungal CFS in terms of antibacterial activity against all of the tested pathogens (*E. coli ATCC^®^ 25922*, *S. dysenteriae*, *A. hydrophila*, and *E. faecalis)*, with all *p*-values < 0.05. These findings support the improved inhibitory zone characteristics of AgNPs, largely due to their direct contact with bacterial membranes via size-dependent mechanisms. These findings are also supported by various other studies claiming antimicrobial efficacy of AgNPs against Gram-negative and Gram-positive bacteria and validating them as future alternatives to current antibacterial agents due to the ever-growing presence of antimicrobial resistance [41,42,43,44,45].

The antibacterial analysis in our study has yielded highly significant results in *p*-values against the Gram-negative bacterial strains *E. coli ATCC^®^ 25922* (*p* = 0.0013) and *S. dysenteriae* (*p* = 0.0059), indicating the ability to cross the outer membranes, unlike CFS, to release ions. Then, in the case of the Gram-positive bacterial strain of *E. faecalis* (*p* = 0.0021), the ability to lyse peptidoglycans, along with the moderate significance of *A. hydrophila* (*p* = 0.0286), correlates with the previously mentioned resistance overcome by the tested AgNP formulas. This wide-spectrum activity has been observed in several studies recording zones of inhibition as 12–20 mm through biosynthesized AgNPs against these bacteria [41,42,46,47].

The statistically significant efficiency of biosynthesized AgNPs as antibacterial agents is also due to the Ag^+^ ions inhibiting microbial electron transport chains and deteriorating deoxyribonucleic acid (DNA) and ribonucleic acid (RNA) through binding. Similarly, Ag^+^ also prevents cell separation by preventing DNA duplication and ions ROS, which are lethal to eukaryotic host cells and bacterial cells [48].

Our study employed the Agar Well Diffusion method to observe parallel antimicrobial activity between biosynthesized AgNPs and a pure CFS of the same strain. However, only the biosynthesized AgNPs from FCG-8 demonstrated remarkable inhibitory potential against *E. coli*, *S. dysenteriae*, *A. hydrophila*, and *E. fecalis*, offering a green alternative to antibiotics amid rising resistance, and the fact that these AgNPs might be used as broad-spectrum antimicrobials has been acknowledged [49,50,51].

## 4. Materials and Methods

The current study was executed at the Microbiology Lab, Department of Biomedical Engineering, Sir Syed University of Engineering and Technology, Karachi, Pakistan; Advanced Research Lab, Dow University of Health Sciences, Karachi, Pakistan; and International Center for Chemical and Biological Sciences, H.E.J. Research Institute of Chemistry, University of Karachi, Pakistan. The synthesis parameters were adopted from previously reported microbial AgNP synthesis protocols and were not subjected to systematic optimization in the present study.

### 4.1. Reidentification of C. glabrata Isolate

A single clinical isolate of *C. glabrata* (FCG-8) was collected and reidentified by using conventional diagnostic standards such as the wet mount technique, Gram staining, germ tube tests, and growth on SDA (CM1002, Merck, Darmstadt, Germany), CHROMagar (CM1007, Oxoid, Hamburg, Germany), and Corn Meal Agar (Tween 80, Merck, Darmstadt, Germany), while this was confirmed by sugar assimilation, sugar fermentation tests, and Rapid^TM^ yeast plus system kits test. Consequently, the reidentified strain of *C. glabrata* was utilized for AgNP biosynthesis.

The strain of *C. glabrata* was cultivated separately in 250 milliliters (mL) of Sabouraud Dextrose Broth and incubated at 37 °C for 48 h in a shaking incubator (Orbital Shaker Incubator, ES-20, Grant Bio, Cambridge, UK, and Orbital Shaker Incubator, Vertical Type, LM-575RD, YIH DER, New Taipei City, Taiwan) at 150 revolutions per minute (rpm). Later, the broth was transferred into a 50 mL Falcon tube and centrifuged (Clinical Centrifuge, DM0636, DLAB, Beijing, China) at 4000 rpm for 30 min. Consequently, the sediment was removed, and the resulting supernatant was gathered and purified with a syringe filter having a pore size of 0.22 micrometer (µm). The supernatant was then kept at 4 °C until it was required again. This is how the CFS solution of the *C. glabrata* isolate was prepared individually. Then, the preparation of a 2 millimolar (mM) AgNO_3_ solution was carried out by taking 0.0339 g of AgNO_3_ (Sigma Aldrich Chemicals, St. Louis, MO, USA), dissolving it in 100 mL of Milli-Q (mQ) water, and placing it in the dark so the reduction process could be carried out [29].

### 4.2. Biosynthesis and Purification of AgNPs

The AgNPs were synthesized by the addition of 10 mL of CFS of *C. glabrata* strain to 90 mL of 2 mM AgNO_3_ solution and keeping this at 150 rpm and 37 °C in a shaking incubator for up to 48 h in a dark environment. A noticeable shift in color from transparent to dark brown suggested that AgNPs might have formed. After the incubation, a 100 mL solution of AgNPs was centrifuged at 4000 rpm for 30 min and then washed thrice with mQ water to remove the remaining supernatant and ensure the purification of AgNPs. Afterward, the AgNPs were put in a sonicator (Ultrasonic Water Bath, Grant Bio, Cambridge, UK) at 37 °C for 30 min to homogenize the AgNPs for further use [52,53].

### 4.3. UV-Vis Spectroscopy

UV-Vis spectrophotometer (MultiSkan SkyHigh Microplate, Spectrophotometer, Thermo Scientific, Waltham, MA, USA) was used to perform characterization of AgNPs. Approximately 5 mL of each sample, i.e., blank solution (mQ water), AgNO_3_ (without CFS), and AgNP solution (CFS of *C. glabrata* along with AgNO_3_), was taken and investigated across wavelengths between 200 and 800 nm [54].

### 4.4. XRD

XRD (Bruker D8 Advance Powder X-Ray Diffractometer, Bruker, Billerica, MA, USA) was used to determine the physicochemical properties and structural nature of biosynthesized AgNPs. The investigation was performed by placing a tiny coating of lyophilized sample taken on a glass slit and then testing it at 40 kilovolts (kV) and 25 milliampere (mA) with angles from 5° to 90°. The Debye–Scherrer formula was used for XRD analysis, expressed in Equation (1):

D = K λ/β Cosθ(1)

where D represents the size of the crystallite, K signifies the shape factor, λ denotes the wavelength of the X-ray, β indicates the broadening of the line at half the peak intensity, and θ refers to the Bragg angle [55].

### 4.5. EDX and SEM

EDX coupled with SEM (Apreo 2 C LoVac Scanning Electron Microscope, Thermofisher Scientific, Waltham, MA, USA) techniques were used to examined the surface characteristics, elemental arrangement, morphology, and dimensions of biosynthesized AgNPs by placing the lyophilized sample, which had a thin coating of ground carbon and was covered in a gold layer, under the microscope [56]. Further, the sizes of the AgNPs were measured using SEM images using ImageJ software. Images were calibrated using the scale bar that was part of the images and isolated individual AgNPs through thresholding. Each AgNP was then outlined either manually or automatically, enabling the software to calculate its dimensions, such as diameter or equivalent circular size. In these images, multiple AgNPs per sample were measured. ImageJ provides a uniform, reproducible, and accurate statistical distribution of AgNP sizes, even for those AgNPs that are irregularly shaped, as seen in SEM images.

### 4.6. Antibacterial Activity of Biosynthesized AgNPs

The antibacterial properties of AgNPs using *C. glabrata* isolate (FCG-8) were determined by comparing the effects of the respective CFS against Gram-negative bacteria such as *E. coli*, *S. dysenteriae*, and *A. hydrophila.* In the same way, the antibacterial potential was also compared against Gram-positive bacteria such as *E. fecalis*. These antimicrobial activities were assessed using the Agar Well Diffusion method with Mueller–Hinton Agar. For the determination of the antibacterial activity of AgNPs, the CFS of the *C. glabrata* isolate and biosynthesized AgNPs from the same *C. glabrata* isolate were examined separately.

Following cultivation in a nutrient broth, the bacterial strains were adjusted to a McFarland index of 0.5. Subsequently, 100 µL of each bacterial strain was placed onto a Mueller–Hinton Agar plate. Additionally, three wells, each 10 mm in diameter, were created on each plate using a sterile cork borer. Then, the wells of each plate were filled with 100 μL of CFS containing AgNPs at a concentration of 1 mg/mL, prepared in mQ water, was used (designated as 1) and 100 µL of AgNPs at the same concentration (designated as 2), which were biosynthesized by FCG-8. A normal saline solution was added to each agar plate as a negative control (designated as C). The plates were subsequently incubated at 37 °C for 24 to 48 h in a Forced Convection Laboratory Incubator (ESCO Isotherm). After incubation, the sizes of the inhibition zones were measured in mm [57,58]. We employed saline (0.9%) as a negative control and a commercially available antibiotic disk, which contains Cefadroxil (a first-generation cephalosporin antibiotic at a concentration of 0.0025 µg/µL with a sensitivity index of less than 20 mm). 

Characterization of the contagious strains (*S. dysenteriae*, *A. hydrophila*, and *E. faecalis*) employed in this research was obtained using conventional and biochemical procedures, and is provided in Tables S1 and S2 of the Supplementary Materials. The validation of the current findings is performed with the results reported in literature and studies [59,60,61,62,63], offering morphological, cultural, and biochemical insights following established diagnosis methods.

### 4.7. Statistical Analysis

The data were analyzed using OriginLab Pro (version 2024a), ImageJ, and SPSS (version 28) professional software. These software enabled the systematic arrangement of data and the generation of graphs and scatter plots that illustrate the size distribution of biosynthesized AgNPs.

## 5. Conclusions

The current study has successfully demonstrated an innovative and environmentally sustainable approach for producing AgNPs using *C. glabrata*, which has emerged as a promising bio-factory for the green synthesis of AgNPs. In this study, the UV-Vis spectroscopy findings indicated that AgNPs formed by a single strain of *C. glabrata* (FCG-8) have a solitary peak, absorbing the most at approximately 430 nm. With XRD, EDX, and SEM, it was verified that crystalline and cubic AgNPs were biosynthesized, with an average crystal size of 21.91 nm, an Ag composition of 29.50%, and AgNP size ranging from 74.96 to 100.40 nm. However, research in this field is still sparse, and the involvement of *C. glabrata* in the biosynthesis of AgNPs remains underexplored compared to other microorganisms. Our study emphasized the potential of this opportunistic yeast for practical applications in nanotechnology, paving the way for new antimicrobial uses. The biologically produced AgNPs exhibited exceptional antibacterial properties against both Gram-positive and Gram-negative bacteria. Therefore, these AgNPs could be developed, commercialized, and utilized in the worldwide healthcare sector to address a range of clinically relevant human pathogens. Nevertheless, additional preclinical investigations are necessary to assess the therapeutic efficacy of these AgNPs. However, the possible genotoxic and cytotoxic impacts of AgNPs on human cells, along with their environmental aggregation, highlight the need for thorough examination. Furthermore, standardized toxicity assessments must be conducted before AgNPs can be recommended for practical or biomedical applications.

## Figures and Tables

**Table 1:** Validation of *C. glabrata* isolate by conventional methods.

Sample Code	Gram Staining Microscopy	SDA Colonial Characteristic	Germ Tube Test	*Candida* CHROM Agar Test	Corn Meal Tween 80 Morphology	Growth at 45 °C	Sugar												
							Assimilation Test							Fermentation Test					
							Dextrose	Sucrose	Lactose	Maltose	Xylose	Trehalose	Galactose	Dextrose	Sucrose	Lactose	Maltose	Trehalose	Galactose
FCG-8	short oval-shaped yeast	smooth and white small colonies	-ve	beige/yellow/brown color	oval, single terminal budding, and not capsulated	-ve	+	−	−	+	−	+	−	+	−	−	−	+	−

**Table 2:** The amount of other elements alongside silver according to the EDX analysis.

Element	Atomic (%)	Atomic (%) Error	Weight (%)	Weight (%) Error
Carbon (C)	19.5	0.7	6.7	0.2
Oxygen (O)	24.7	1.3	11.3	0.6
Sodium (Na)	21.9	0.4	14.3	0.3
Chlorine (Cl)	21.5	0.5	21.7	0.5
Silver (Ag)	9.6	0.4	29.5	1.1
Lead (Pb)	2.8	0.4	16.5	2.2

**Table 3:** Antimicrobial activity of CFS, AgNPs, and controls against clinical pathogens. Values are presented as mean ± standard error of the mean calculated from three independent replicates (n = 3). Zones of inhibition were measured in millimeters (mm). Cefadroxil concentration was 0.0025 microgram/microliter (µg/µL).

Sample	Antimicrobial Treatment	*Escherichia coli* (*ATTC^®^ 25922*)	*Shigella* *dysenteriae*	*Aeromonas hydrophila*	*Enterococcus* *faecalis*
FCG-8	CFS (zone of inhibition, mm)	0.0 ± 0.00	0.0 ± 0.00	7.7 ± 0.33	5.7 ± 0.66
	AgNPs (zone of inhibition, mm)	18.7 ± 0.66	13.0 ± 1.00	10.7 ± 0.66	13.3 ± 0.33
Negative control	0.9% normal saline	0.0 ± 0.00	0.0 ± 0.00	0.0 ± 0.00	0.0 ± 0.00
Positive control	Cefadroxil * (zone of inhibition, mm)	26.3 ± 0.66	20.0 ± 0.00	21.3 ± 0.66	18.7 ± 0.66

* First-generation cephalosporin antibiotic with a sensitivity index less than 20 mm.

## Data Availability

The original contributions presented in this study are included in the article/Supplementary Materials. Further inquiries can be directed to the corresponding authors.

## Supplementary Materials

The following supporting information can be downloaded at https://www.mdpi.com/article/10.3390/ijms27031263/s1. The results shown in Tables S1 and S2 are in agreement with previously reported literature and studies [59,60,61,62,63].
