# Supplementary material for: Biogenesis of Candida glabrata-Mediated Silver Nanoparticles: Characterization and Antibacterial Effectiveness Against Human Pathogenic Bacteria
Source: Int J Mol Sci. 2026 Jan 27;27(3):1263. doi: 10.3390/ijms27031263 (PMC12897783; doi:10.3390/ijms27031263)
Supplement: Supplementary file 1 [file ijms-27-01263-s001.zip › ijms-4084164-supplementary.pdf]

Table S1. Characterization of bacterial strains using conventional methods.

Organisms	Morphological characteristics in differential media				
	**Gram staining**	**Media**	**Characteristics**	**Media**	**Characteristics**
Shigella dysenteriae	*Bacillus* rod (pink color)	MacConkey agar	colorless (non-lactose fermenting)	*Salmonella Shigella* agar	colorless colonies
Aeromonas hydrophila	*Bacillus* rod (pink color)	MacConkey agar	lactose non-fermenting	blood agar	β-hemolytic
Enterococcus faecalis	*Cocci* round (purple color)	Bile Esculin agar	blackening (positive)	blood agar	small, gray, non-hemolytic or α-hemolytic colonies

Table S2. Characterization of bacterial strains using biochemical methods.

Organisms	Biochemical test							
	**Lactose test**	**Indole test**	**Urease test**	**Citrate test**	**TSI test**	**Oxidase**	**Catalase**	**Grows in 6.5% NaCl broth (tolerance test)**
Shigella dysenteriae	non-fermenter	-ve	-ve	-ve	alkaline/acid, no gas, no H_2S	-ve	-ve	NA
Aeromonas hydrophila	non-fermenter	+ve	-ve	+ve	alkaline/acid, no H_2S	+ve	+ve	NA
Enterococcus faecalis	NA	NA	NA	NA	NA	NA	-ve	Growth in 6.5% NaCl (positive)

Here, NA = Not available, +ve = Positive, -ve = Negative, TSI = Triple sugar iron, NaCl = sodium chloride, and H_2S = hydrogen sulfide.
